# Risks and rewards of increasing patient access to medical records in clinical ophthalmology using OpenNotes

**DOI:** 10.1038/s41433-021-01775-9

**Published:** 2021-10-05

**Authors:** Jake E. Radell, Jasmine N. Tatum, Chen-Tan Lin, Richard S. Davidson, Jonathan Pell, Amber Sieja, Albert Y. Wu

**Affiliations:** 1 Department of Ophthalmology, Icahn School of Medicine at Mount Sinai, New York, NY USA; 2 Department of Psychiatry, Stanford University School of Medicine, Stanford, CA USA; 3 Department of Medicine, University of Colorado School of Medicine, Aurora, CO USA; 4 Department of Ophthalmology, University of Colorado School of Medicine, Aurora, CO USA; 5 Department of Ophthalmology, Stanford University School of Medicine, Stanford, CA USA

**Keywords:** Health care, Business and industry

## Abstract

**Background:**

The implementation of OpenNotes and corresponding increase in patient access to medical records requires thorough assessment of the risks and benefits of note-sharing. Ophthalmology notes are unique among medical records in that they extensively utilize non-standardized abbreviations and drawings; they are often indecipherable even to highly-educated clinicians outside of ophthalmology. No studies to date have assessed ophthalmologist perceptions of OpenNotes.

**Methods:**

A cross-sectional study was conducted from 4/28 to 5/12/2016. A survey was distributed to 30 clinicians (25 ophthalmologists, three optometrists, two nurses) in the University of Colorado’s Department of Ophthalmology to evaluate provider attitudes towards granting patients access to online medical records.

**Results:**

Many clinicians felt patients would have difficulty understanding their records and may be unnecessarily alarmed or offended by them. Some clinicians worried their workload would increase and feared having to change the way they document. Perceived benefits of OpenNotes included improving patient understanding of their medical conditions, strengthening patient–physician trust, and enhancing patient care. Many perceived risks and benefits of note-sharing were associated with conceptions of the ideal clinician–patient relationship.

**Conclusions:**

Clinicians in ophthalmology perceived both benefits and consequences of increasing patient access to ophthalmic records, and there were significant correlations between these perceptions and their conceptions of the clinician–patient relationship. This is the first study to assess potential ophthalmology provider attitudes toward sharing ophthalmic records. Although limited in sample size and power, this study demonstrates some ways patient-accessible ophthalmic records can affect the clinical practice of ophthalmology and emphasizes the unique challenges of OpenNotes in ophthalmology.

## Introduction

Patients’ ability to access their medical records has been a key component of the evolution of a more patient-centered healthcare system, and development of programs that enable easy access to electronic medical records has facilitated this evolution. In particular, the “OpenNotes” program has grown rapidly over the past several years, allowing patients to view the notes written by their providers via an online portal. Per their website, OpenNotes is “an international movement committed to spreading and studying the effects of transparent communication among patients, families and clinicians”. In practice, adoption of OpenNotes by healthcare systems involves dramatically increasing the accessibility of electronic medical record notes to patients using patient-facing online portals. OpenNotes is not in itself a method of note-sharing, but rather, a movement pushing for health systems to use existing electronic medical record-based tools to openly share notes with patients. As of 2020, over 50 million patients in the United States had access to their medical records through patient portals [1]. This number should further increase following the 4/5/2021 milestone set by the 21st Century Cures Act in the US [2].

Several studies have shown that increasing patient access to medical records can enhance doctor–patient communication, reduce errors, and improve healthcare quality [3–7]. Despite these potential benefits, enabling patients to view their medical records may also have negative consequences. Preliminary studies found that access to medical records has the potential to unnecessarily concern or confuse patients, and many clinicians are concerned that such access will increase their workload if patients contact the office with questions about their records [8–18]. Additionally, there is concern that clinicians may change the way they document if they know the records will be viewed by patients, potentially rendering them less useful to healthcare professionals and patients alike [18, 19]. However, more recent studies indicate that after the implementation of note-sharing, clinician concerns over an increase in workload significantly decrease and most clinicians are in favor of sharing notes with patients [20–22]. In fact, the vast majority of clinicians surveyed after using OpenNotes agreed that note-sharing is a good idea and increases patient engagement, with 44% of clinicians changing their opinion on OpenNotes from negative to positive after implementation [21, 22].

The risks and benefits of patient access to online medical records have been studied previously in several fields of medicine, but not in ophthalmology. Increased access to medical records poses unique challenges in ophthalmology for multiple reasons. First, clinical ophthalmology has a complex workflow that involves various medical personnel, including technicians, photographers, nurses, optometrists, and physicians. Evaluations typically require numerous procedures and tests involving different imaging modalities and measurement devices at each visit. Most of these modalities and measurements yield results that are not readily interpretable by patients. The multidimensional nature of clinical ophthalmology considerably complicates medical records and may make them less decipherable to patients. Second, clinical ophthalmology is a fast-paced, high-volume outpatient field. With hundreds of patients to see each week, clinicians may have less time to explain their medical notes to patients. Third, ophthalmology notes are written using a large lexicon and a multitude of abbreviations (both standardized and unstandardized) [23–27]. It can be difficult for non-ophthalmologist physicians to understand ophthalmology notes, let alone patients without medical training. While there is an argument that ophthalmologists should work to increase readability of their documentation rather than continue to use often unintelligible acronyms, a movement towards such a large change in documentation patterns has not yet gained strength and would certainly take years to implement. In the meantime, ophthalmology notes remain incomprehensible to the vast majority of people. Lastly, ophthalmology is a visually-oriented field and many practitioners rely heavily on drawings and annotations created with visual templates. Not only are these drawings routinely left out from online medical notes, but, if they are included, they are often uninterpretable to the untrained eye. Despite the difficulties of interpreting ophthalmology notes, the only known study surveying ophthalmology patients on this issue found that patients strongly desired access to their ophthalmology notes and believed such access would benefit their care [28]. This finding is in keeping with those of non-ophthalmology studies showing that patients perceive OpenNotes as beneficial to their care and their health [29, 30]. In the context of recent legislative changes and well-documented patient desire to access notes, it is essential that the medical community gain a greater understanding of clinician perceptions of note-sharing, particularly in unique fields such as ophthalmology.

Here we administered a survey to faculty ophthalmologists at the Department of Ophthalmology at the University of Colorado Hospital to better understand the unique implications of encouraging patient access to electronic ophthalmic medical records.

## Methods

From April 28 to May 12, 2016, a questionnaire measuring perceived risks and benefits of increasing patient access to online medical records was distributed by e-mail to 30 clinicians including ophthalmologists, nurses, and optometrists, in the Department of Ophthalmology at University of Colorado. Responses were accepted only before implementation of OpenNotes. The questionnaire included questions assessing respondents’ beliefs about clinician–patient relationships. A composite score assessed each clinicians’ general perceptions of sharing medical records with patients and was calculated by averaging the responses to the questionnaire. The questionnaire had been used in previous studies and had been reported to have strong reliability and validity [18, 31, 32]. No identifying information was obtained. Both University of Colorado and Mount Sinai approved the study, which was exempted from Institutional Review Board approval.

The questionnaire also gathered demographic information about respondents including age, gender, years in practice, highest degree earned, and specialty.

A Spearman’s correlation test (alpha = 0.05) was used to determine whether demographic characteristics or conceptions of the clinician–patient relationship were associated with perceived risks or benefits of increasing patient access to medical records.

## Results

### Participant characteristics

Out of 30 clinicians recruited, 29 participated in the study (96.7% response rate). Of the 29 participants for whom data was available, 9 (31%) were female and mean age was 47.18 (SD 10.47; range 31–66). Twenty-four of 29 clinicians (82.8%) had MD degrees and identified themselves as “ophthalmologists” (Table 1). More than half (55%) of clinicians had been in practice for over 15 years, and all clinicians reported practicing for more than 3 years. Two clinicians (6.9%) reported routinely sending copies of clinical notes to patients.

**Table 1:** Respondent characteristics (*N* = 29).

Characteristic	Frequency (%)
Age, mean ± SD [range]^a^	47.2 ± 10.5 [31–66]
Gender	
Male	20 (69)
Female	9 (31)
Clinician title	
Physician	24 (82.8)
Other clinician	5 (17.2)
Years practicing	
Under 1 year	0 (0)
Between 1 and 3 years	0 (0)
Between 3 and 5 years	1 (3.4)
Between 5 and 10 years	8 (27.6)
Between 10 and 15 years	4 (13.8)
Between 15 and 20 years	6 (20.7)
Between 20 and 30 years	3 (10.3)
Over 30 years	7 (24.1)
Practice in a clinical setting	20 (100)
Routinely sends copies of clinical notes to patients	2 (6.9)

^a^Data regarding age was available for 28 of the 29 participants. One participant reported that he was 10 years old and had been a practicing Ophthalmologist for 60 years. Due to this inconsistency and suspected error in reporting, he was excluded from all data analysis involving age.

### Perceived risks and benefits of using patient-accessible online medical records

**Fig. 1:**
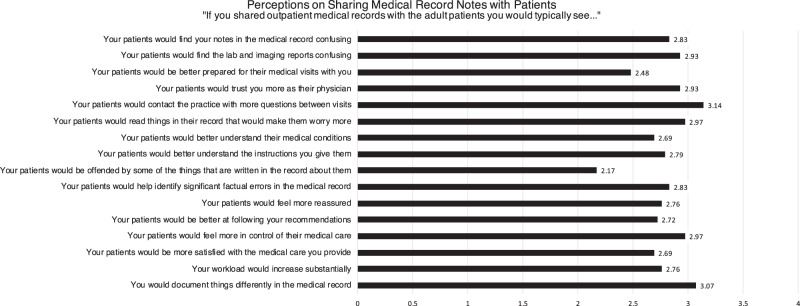
Perceived risks and benefits of using patient-accessible online ophthalmic medical records (Mean). Responses are graded on a Likert scale from 1 (strongly disagree) to 4 (strongly agree). The length of each bar indicates the mean response for each question.

Figure 1 summarizes mean responses on a Likert Scale (from 1 to 4: strongly disagree to strongly agree) to questions assessing general expectations of clinicians regarding encouraging patients to access their ophthalmology medical records online.

All clinicians surveyed identified potential negative consequences. Many believed that patients would find the medical record confusing and have a hard time interpreting test and imaging reports (69% and 79.3% respectively). Most clinicians (75.9%) believed patients would worry more if they could access their medical records, and 89.7% predicted that their patients would be offended by some things written in their record. 82.8% were concerned that patients would contact their practice with more questions between visits, and 86.2% were concerned about having to document differently in the medical record. 55.2% of clinicians were worried their workload would substantially increase. Although many clinicians expressed concern about OpenNotes, survey respondents also believed note-sharing could be beneficial. 48.3% of clinicians believed patients would be better prepared for medical visits, and 82.8% believed sharing medical records would increase patient–physician trust. Many clinicians felt that their patients would better understand their medical conditions (65.6%), better comprehend instructions given (72.4%), and be better able to follow medical recommendations (65.5%). 79.4% believed patients would be able to help identify significant factual errors in the medical record, and 89.7% thought patients would feel more in control of their medical care. When asked if sharing medical records would increase patient satisfaction with medical care, 69% agreed that it would.

### Assessing clinician–patient relationships

**Fig. 2:**
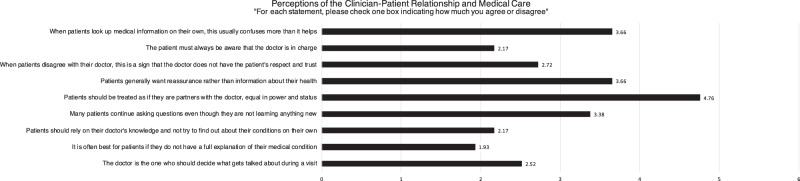
Perceptions of the clinician–patient relationship (Mean). Responses are graded on a Likert scale from 1 (strongly disagree) to 6 (strongly agree).

Figure 2 summarizes mean responses on a Likert Scale (from 1 to 4: strongly disagree to strongly agree) to questions assessing attitudes regarding the clinician–patient relationship. 89.7% felt that patients should be treated as partners with the doctor, equal in power and status. All but one clinician disagreed that the patient must always be aware the doctor is in charge (96.5%), and 75.9% also disagreed that the doctor should be the one deciding what gets discussed during visits. 20.7% of clinicians felt patient-doctor disagreements are a sign of patients’ disrespect and mistrust. 93.1% felt it was best if patients have full explanations of their medical conditions, yet when asked if patients generally want reassurance rather than information about their health, 55.2% agreed that patients preferred reassurance over information. 55.2% felt that when patients look up medical information on their own, it usually confuses them more than it helps, yet 96.6% disagreed that patients should rely on their doctor’s knowledge and refrain from researching their conditions. 51.7% felt that many patients continued asking questions even though they are not learning anything new.

### Factors influencing perceptions

Clinicians’ responses to potential risks and benefits of providing patients with access to online ophthalmic medical records, as summarized by composite score of clinician perspectives, were not significantly associated with gender, age, years in practice, or the clinician’s role.

Some perceptions of the clinician–patient relationship were found to be significantly associated with perceived benefits or risks of increasing patient access to online medical record (Tables 2–4). The degree to which doctors felt physicians should decide what gets discussed during a medical visit and the degree to which they believed it was best if patients did not have a full explanation of their records were both negatively correlated (*p* < 0.01, *R* = −0.519; *p* < 0.05, *R* = −0.430) with the belief that patients would better understand instructions if they had access to their medical records. The extent to which clinicians felt patient–physician disagreements are signs of disrespect/mistrust was negatively correlated with fear of an increase in workload (*p* < 0.05, *R* = −0.465). The extent to which clinicians felt patient–physician disagreements are signs of disrespect/mistrust was positively correlated with feeling the patient would be better prepared for visits (*p* < 0.05, *R* = +0.434), better understand medical conditions (*p* < 0.01, *R* = +0.525), and better understand instructions given to them (*p* < 0.05, *R* = +0.443). The degree to which clinicians believed patients should realize the doctor is always in charge was negatively correlated with worry that patients would be offended by what is written in medical records (*p* < 0.05, *R* = −0.385). Finally, the degree to which physicians felt patients who look up information on their own are more often confused than helped was negatively correlated with feeling the patient would be better prepared for medical visits (*p* < 0.01, *R* = −0.492).

**Table 2:** Correlations between perceptions of the clinician–patient relationship and perceptions of sharing online medical records with patients.

Outcomes of sharing online medical records	Spearman’s	The doctor is the one who should decide what gets talked about during a visit	It is often best for patients if they do not have a full explanation of their medical condition.	Patients should rely on their doctor’s knowledge and not try to find out about their conditions on their own.	Many patients continue asking questions even though they are not learning anything new.	Patients should be treated as if they are partners with the doctor, equal in power and status.	Patients generally want reassurance rather than information about their health.	When patients disagree with their doctor, this is a sign that the doctor does not have the patient’s respect and trust.	The patient must always be aware that the doctor is in charge.	When patients look up medical information on their own, this usually confuses more than it helps.
Your patients would find your notes in the medical record confusing	Correlation Coefficient	−0.029	0.333	0.217	0.11	0.002	0.309	−0.2	−0.231	0.274
	Sig. (two-tailed)	0.883	0.078	0.259	0.57	0.993	0.102	0.299	0.228	0.15
Your patients would find the lab and imaging reports confusing	Correlation Coefficient	0.051	0.018	0.189	0.24	−0.112	0.051	−0.269	−0.199	0.163
	Sig. (two-tailed)	0.792	0.926	0.326	0.21	0.563	0.795	0.159	0.302	0.397
Your patients would be better prepared for their medical visits with you	Correlation Coefficient	−0.204	−0.291	−0.059	−0.233	0.011	−0.344	**0.434**^**a**^	−0.051	**−0.492**^**b**^
	Sig. (two-tailed)	0.288	0.125	0.759	0.224	0.955	0.068	0.019	0.794	0.007
Your patients would trust you more as their physician	Correlation Coefficient	−0.109	−0.292	−0.081	−0.131	0.087	−0.281	−0.155	−0.127	−0.36
	Sig. (two-tailed)	0.572	0.124	0.675	0.498	0.655	0.14	0.421	0.51	0.055
Your patients would contact the practice with more questions between visits	Correlation Coefficient	0.232	0.037	0.267	0.065	−0.117	0.051	−0.208	−0.135	0.093
	Sig. (two-tailed)	0.225	0.848	0.162	0.739	0.547	0.795	0.279	0.485	0.632

Bold = statistically significant.

^a^Correlation is significant at the 0.05 level (two-tailed).

^b^Correlation is significant at the 0.01 level (two-tailed).

**Table 3:** Correlations between perceptions of the clinician–patient relationship and perceptions of sharing online medical records with patients.

Outcomes of sharing online medical records	Spearman’s	The doctor is the one who should decide what gets talked about during a visit	It is often best for patients if they do not have a full explanation of their medical condition.	Patients should rely on their doctor’s knowledge and not try to find out about their conditions on their own.	Many patients continue asking questions even though they are not learning anything new.	Patients should be treated as if they are partners with the doctor, equal in power and status.	Patients generally want reassurance rather than information about their health.	When patients disagree with their doctor, this is a sign that the doctor does not have the patient’s respect and trust.	The patient must always be aware that the doctor is in charge.	When patients look up medical information on their own, this usually confuses more than it helps.
Your patients would read things in their records that would make them worry more	Correlation Coefficient	0.112	0.139	−0.027	0.226	−0.004	0.125	−0.047	−0.193	0.321
	Sig. (two-tailed)	0.562	0.471	0.89	0.238	0.985	0.518	0.811	0.316	0.09
Your patients would better understand their medical conditions	Correlation Coefficient	0.01	0.034	0.145	0.136	−0.078	−0.154	**0.525**^**a**^	0.305	−0.22
	Sig. (two-tailed)	0.96	0.863	0.454	0.48	0.689	0.426	0.003	0.108	0.251
Your patients would better understand the instructions you give them	Correlation Coefficient	**−0.519**^**a**^	**−0.430**^**b**^	−0.09	−0.084	0.289	−0.064	**0.443**^**b**^	−0.009	−0.07
	Sig. (two-tailed)	0.004	0.02	0.641	0.666	0.128	0.741	0.016	0.961	0.718
Your patients would be offended by some of the things that are written in the record about them	Correlation Coefficient	0.086	0.048	−0.035	−0.127	0.084	−0.119	−0.101	**−0.385**^**b**^	−0.151
	Sig. (two-tailed)	0.658	0.807	0.858	0.511	0.665	0.538	0.601	0.039	0.434
Your patients would help identify significant factual errors in the medical record	Correlation Coefficient	−0.231	−0.052	−0.024	−0.295	0.269	−0.092	−0.04	−0.042	−0.088
	Sig. (two-tailed)	0.227	0.789	0.902	0.12	0.158	0.636	0.835	0.827	0.648

Bold = statistically significant.

^a^Correlation is significant at the 0.01 level (two-tailed).

^b^Correlation is significant at the 0.05 level (two-tailed).

**Table 4:** Correlations between perceptions of the clinician–patient relationship and perceptions of sharing online medical records with patients.

Outcomes of sharing online medical records	Spearman’s	The doctor is the one who should decide what gets talked about during a visit	It is often best for patients if they do not have a full explanation of their medical condition.	Patients should rely on their doctor’s knowledge and not try to find out about their conditions on their own.	Many patients continue asking questions even though they are not learning anything new.	Patients should be treated as if they are partners with the doctor, equal in power and status.	Patients generally want reassurance rather than information about their health.	When patients disagree with their doctor, this is a sign that the doctor does not have the patient’s respect and trust.	The patient must always be aware that the doctor is in charge.	When patients look up medical information on their own, this usually confuses more than it helps.
Your patients would feel more reassured	Correlation Coefficient	0	−0.047	−0.007	−0.021	−0.081	−0.263	0.024	0.181	−0.248
	Sig. (two-tailed)	1	0.809	0.972	0.914	0.675	0.167	0.903	0.348	0.195
Your patients would be better at following your recommendations	Correlation Coefficient	−0.045	−0.064	−0.145	0.067	−0.073	−0.043	0.231	0.104	−0.025
	Sig. (two-tailed)	0.817	0.742	0.452	0.729	0.708	0.824	0.228	0.592	0.898
Your patients would feel more in control of their medical care	Correlation Coefficient	−0.209	−0.035	−0.004	−0.362	−0.168	−0.025	0.166	0.111	−0.339
	Sig. (two-tailed)	0.278	0.859	0.983	0.054	0.384	0.898	0.388	0.567	0.072
Your patients would be more satisfied with the medical care you provide	Correlation Coefficient	0.125	−0.033	−0.157	0.005	−0.091	0.042	0.094	0.248	−0.114
	Sig. (two-tailed)	0.517	0.863	0.415	0.981	0.64	0.831	0.627	0.195	0.554
Your workload would increase substantially	Correlation Coefficient	0.265	0.127	0.125	0.028	−0.092	0.248	**−0.465**^**a**^	−0.28	0.298
	Sig. (two-tailed)	0.165	0.512	0.519	0.886	0.636	0.195	0.011	0.142	0.117
You would document things differently in the medical record	Correlation Coefficient	0.153	0.286	0.176	0.04	−0.088	0.178	−0.235	−0.301	0.032
	Sig. (two-tailed)	0.427	0.133	0.361	0.838	0.65	0.357	0.22	0.113	0.868

Bold = statistically significant.

^a^Correlation is significant at the 0.05 level (two-tailed).

## Discussion

The results of this study suggest that implementation of patient-accessible medical records may be uniquely challenging in ophthalmology. The main obstacle appears to be patient comprehension of medical records. Nearly 80% of University of Colorado ophthalmology clinicians believed patients would have difficulty interpreting ophthalmic records. This majority of clinicians is especially striking when compared to a previous study at University of Colorado showing that only 14% of Cardiology clinicians believed patients would have difficulty interpreting online cardiovascular medical records [18]. The perceived decrease in patient comprehension could be attributed to factors that make ophthalmic medical records difficult to understand, including their multidimensional nature, complicated and unstandardized abbreviations, and reliance on visual drawings and annotations.

Regardless of the source of this perceived comprehension barrier, it is imperative that ophthalmologists work to reduce this barrier in the era of legally mandated note-sharing which began on 4/5/2021. Patients will be reading ophthalmologist notes moving forward, so it falls to the ophthalmologist community to work toward making their notes comprehensible to patients and clinical colleagues without an abbreviation dictionary on hand. Technology may be of help in this process, as with electronic medical record systems approaching universal adoption, the tools for automatically translating ophthalmic abbreviations should become more widely available and routinely used.

Although this study corroborates previous research in indicating OpenNotes’ potential to improve clinician–patient communication, patient adherence, and patient empowerment, it is unclear how these benefits will manifest in ophthalmology given the large comprehension barrier. Additionally, clinicians surveyed perceived a variety of potential drawbacks of sharing medical records with patients, including increasing patient confusion, worry, and stress, increasing clinician workload, and changing the way clinicians document encounters, potentially jeopardizing the integrity and usefulness of medical records. Although, these concerns have been echoed by clinicians in other departments, the percentage of clinicians who feared these negative consequences was greater in Ophthalmology, suggesting that ophthalmology is distinct from other specialties with respect to implementation of patient-accessible medical records.

Several perceptions surrounding physician-patient relationships were associated with ideas about increasing accessibility of medical records. Clinicians who viewed patient–physician arguments as signs of mistrust saw more benefits of notes-sharing; they were significantly less likely to worry about increases in their workload, and more likely to believe patients would be better prepared for visits and better understand their medical conditions and instructions. In contrast, there was a correlation between physicians believing the doctor should always be in charge and physicians seeing negative consequences of note-sharing. Those who believed they should be in charge were significantly more concerned that patients would be offended by note contents. These correlations suggest that physician perceptions regarding the doctor–patient relationship and perceptions regarding patient access to medical records are intimately related. Changing access to patient medical records could alter the clinician–patient relationship, an important pillar of clinical ophthalmology.

This study is limited primarily by small sample size, which limits statistical power. Additionally, because this study only sampled the perceptions of clinicians, not patients, it is unable to assess some potential benefits and risks that note-sharing might have in ophthalmology. Clinician and patient perspectives on whether patients should have access to medical records are often discordant; it is imperative to not only understand how these perspectives diverge, but why.

Importantly, the beliefs assessed in this study were recorded in 2016, before the clinicians began using OpenNotes. Ideally, perceptions about OpenNotes would be assessed before and after its implementation, but this was not feasible here. If surveyed after using OpenNotes, it is likely that some ophthalmologist concerns would be assuaged, while others may be magnified. In 2021, after 5 years of use of OpenNotes, clinical leadership at University of Colorado reports that no significant concerns have been raised by patients despite the fears of the ophthalmologists surveyed in 2016 prior to OpenNotes implementation (Chen-Tan Lin, MD, e-mail communication, June 2021). Future studies should assess patient comprehension of online ophthalmic records and examine how perceptions of note-sharing change after ophthalmologists become accustomed to patient-accessible records. A growing body of literature indicates that after implementation of OpenNotes, clinicians generally view note-sharing as a net positive [1, 20–22], but is important that this question is explored in the field of ophthalmology specifically.

Despite its limitations, this study offers important insights on how increasing patient access to medical records may affect healthcare delivery and doctor–patient relationships. By identifying provider-perceived risks and benefits of providing patients access to online ophthalmic records, this study sets the foundation for future investigation on how such unprecedented access can impact the patient–physician relationship, patient trust, clinical outcomes, and the ophthalmologist’s practice. Finally, this study suggest that ophthalmology clinicians may have fears and concerns regarding the implementation of OpenNotes that differ significantly from those of other clinicians. As such, health systems implementing OpenNotes should recognize the unique challenges that note-sharing poses in ophthalmology, educate ophthalmology providers on the benefits of note-sharing, and collaborate with these providers to work toward the goal of making notes more comprehensible to patients.

## Summary

### What was known before

- The recent development and use of informational programs that facilitate patients’ access to online medical records has made it imperative to uncover the potential risks and benefits of such programs in the field of ophthalmology.
- Ophthalmology notes are unique among medical notes in that they extensively utilize non-standardized abbreviations and drawings by physicians in a way that is often indecipherable even to highly-educated clinicians outside of ophthalmology.

### What this study adds

- Ophthalmology clinicians perceive many benefits to note-sharing but also have fears about OpenNotes that differ significantly from those of other clinicians.
- The unique nature of ophthalmological documentation may augment challenges of OpenNotes implementation in ophthalmology.
